# The effect of cardiac resynchronization without a defibrillator on morbidity and mortality: an individual patient data meta‐analysis of COMPANION and CARE‐HF

**DOI:** 10.1002/ejhf.2524

**Published:** 2022-05-22

**Authors:** John G.F. Cleland, Michael R. Bristow, Nicholas Freemantle, Brian Olshansky, Daniel Gras, Leslie Saxon, Luigi Tavazzi, John Boehmer, Stefano Ghio, Arthur M. Feldman, Jean‐Claude Daubert, David de Mets

**Affiliations:** ^1^ Robertson Centre for Biostatistics & Clinical Trials University of Glasgow & National Heart & Lung Institute, Imperial College London UK; ^2^ University of Colorado Cardiovascular Institute, Aurora and Boulder Aurora CO USA; ^3^ Institute of Clinical Trials and Methodology University College London London UK; ^4^ University of Iowa, Iowa City & Mercy Hospital ‐ North Iowa Mason City IA USA; ^5^ Hopital Prive du Confluent Nantes France; ^6^ Keck School of Medicine University of Southern California Los Angeles CA USA; ^7^ Maria Cecilia Hospital – GVM Care &Research Cotignola Italy; ^8^ Penn State Hershey Medical Center Hershey PA USA; ^9^ Fondazione IRCCS Policlinico S. Matteo Pavia Italy; ^10^ Lewis Katz School of Medicine at Temple University Philadelphia PA USA; ^11^ University of Rennes Rennes France; ^12^ University of Wisconsin School of Medicine and Public Health Madison WI USA

**Keywords:** Heart failure, Individual patient data meta‐analysis, Cardiac resynchronization therapy, Mortality, Body surface area, Sex

## Abstract

**Aims:**

Cardiac resynchronization therapy (CRT) reduces morbidity and mortality for patients with heart failure, reduced left ventricular ejection fraction, QRS duration >130 ms and in sinus rhythm. The aim of this study was to identify patient characteristics that predict the effect, specifically, of CRT pacemakers (CRT‐P) on all‐cause mortality or the composite of hospitalization for heart failure or all‐cause mortality.

**Methods and results:**

We conducted an individual patient data meta‐analysis of the Comparison of Medical Therapy, Pacing, and Defibrillation in Heart Failure (COMPANION) and Cardiac Resynchronization‐Heart Failure (CARE‐HF) trials. Only patients assigned to CRT‐P or control (*n* = 1738) were included in order to avoid confounding from concomitant defibrillator therapy. The influence of baseline characteristics on treatment effects was investigated. Median age was 67 (59–73) years, most patients were men (70%), 68% had a QRS duration of 150–199 ms and 80% had left bundle branch block. Patients assigned to CRT‐P had lower rates for all‐cause mortality (hazard ratio [HR] 0.68, 95% confidence interval [CI] 0.56–0.81; *p* < 0.0001) and the composite outcome (HR 0.67, 95% CI 0.58–0.78; *p* < 0.0001). No pre‐specified characteristic, including sex, aetiology of ventricular dysfunction, QRS duration (within the studied range) or morphology or PR interval significantly influenced the effect of CRT‐P on all‐cause mortality or the composite outcome. However, CRT‐P had a greater effect on the composite outcome for patients with lower body surface area and those prescribed beta‐blockers.

**Conclusions:**

Cardiac resynchronization therapy‐pacemaker reduces morbidity and mortality in appropriately selected patients with heart failure. Benefits may be greater in smaller patients and in those receiving beta‐blockers. Neither QRS duration nor morphology independently predicted the benefit of CRT‐P.

Clinical Trial Registration: COMPANION, NCT00180258; CARE‐HF, NCT00170300.

## Graphical abstract

Potential substrates for and mechanisms of action for cardiac resynchronization therapy pacemakers (CRT‐P). Kaplan–Meier estimates for all‐cause mortality (hazard ratio 0.68, 95% confidence interval [CI] 0.56–0.81; *p* < 0.0001) and time to composite of first hospitalization for heart failure or all‐cause mortality (hazard ratio 0.67, 95% CI 0.58–0.78; *p* < 0.0001). No heterogeneity was identified in the effect of CRT‐P on mortality amongst trial participants. CRT‐P had greater effects on the composite outcome (hospitalization for heart failure or all‐cause mortality) for those taking beta‐blockers and patients of smaller stature. Although women are, on average, smaller than men, no interaction between CRT‐P and sex was observed. Similar trends were observed when only men were included in the analysis. These analyses suggest that stature rather than sex is associated with the effect of CRT‐P on outcome. LBBB, left bundle branch block.

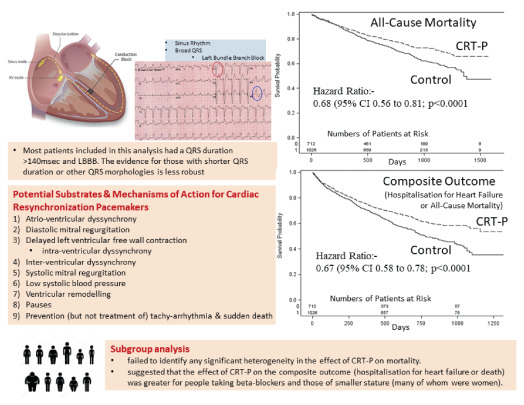

## Introduction

The COMPANION and CARE‐HF trials established the efficacy of cardiac resynchronization therapy (CRT) for patients with moderate or severe symptoms of heart failure, a reduced left ventricular ejection fraction (LVEF), in sinus rhythm and a prolonged QRS duration.^1^, ^2^ Most subsequent trials compared CRT defibrillators (CRT‐D) to implantable cardioverter defibrillators (ICD) in patients with milder symptoms, in order to investigate the incremental benefit of CRT‐D compared to an ICD.^3^, ^4^ COMPANION and CARE‐HF are the only large randomized trials that compared the effects of CRT pacemakers (CRT‐P) to a control group receiving no device and, therefore, the benefits of adding solely CRT‐P to pharmacological therapy.

Controversy persists over the importance of QRS morphology and QRS duration as selection criteria for CRT.^5^ Guidelines emphasize the importance of both QRS morphology and duration, reserving the strongest recommendation for patients with left bundle branch block (LBBB) and QRS duration >150 ms.^6^, ^7^, ^8^, ^9^ However, these recommendations are based predominantly on trials comparing CRT‐D to ICD. Although QRS duration was used to select patients in all the landmark trials of CRT, QRS morphology was an inclusion criterion for none and a pre‐specified subgroup in only two.^1^, ^10^

Accordingly, we investigated the relationships between clinical variables, including QRS morphology and duration, and their ability to predict the benefits of CRT‐P compared to pharmacological therapy alone in an individual patient data (IPD) meta‐analysis of COMPANION and CARE‐HF.

## Methods

The chief investigators for COMPANION (MRB) and CARE‐HF (JGFC) proposed an IPD analysis of these two trials. Medtronic and Boston Scientific facilitated the analyses by providing data‐sharing agreements but provided no other support.

COMPANION aimed to enrol 2200 patients, with 440 patients assigned to the control arm, 880 to CRT‐P and 880 to CRT‐D.^11^ The primary endpoint was the composite of hospitalization for any reason (other than elective device implantation) or all‐cause mortality, with a target of 1000 such events. CARE‐HF aimed to enrol 800 patients with equal numbers randomized to control and CRT‐P.^12^, ^13^ The primary endpoint was the composite of unplanned hospitalization for a major cardiovascular event or all‐cause mortality. All‐cause mortality and the composite of hospitalization for heart failure or all‐cause mortality were secondary endpoints in both trials. Investigators were not blind to treatment allocation but members of the endpoint‐adjudicating committees were.

COMPANION was conducted exclusively in the USA and CARE‐HF in Western Europe but inclusion criteria were otherwise similar.^11^, ^12^ Both trials enrolled patients who, despite pharmacological therapy, had moderate or severe symptoms of heart failure, an LVEF ≤35%, an increased left ventricular (LV) end‐diastolic dimension, were in sinus rhythm and had a QRS duration of ≥120 ms. QRS morphology was not an inclusion criterion for either trial and was a pre‐specified subgroup analysis only for COMPANION. Both trials excluded patients who were not in sinus rhythm or who required pacing or an ICD according to then contemporary guidelines, which was prior to publication of most landmark trials of ICDs. There were some differences between COMPANION and CARE‐HF. COMPANION required a PR interval >150 ms in order to increase the probability of biventricular capture; a requirement that may have enriched the population with patients more likely to benefit from atrio‐ventricular resynchronization.^14^, ^15^ For COMPANION, patients were required to have had a hospital admission or equivalent for the treatment of heart failure between 1 and 12 months prior to enrolment but were excluded if the hospitalization occurred within less than 1 month. Neither hospitalization criterion was required for CARE‐HF. Heart failure had to be of at least 6‐month duration in COMPANION but only 6 weeks in CARE‐HF. In CARE‐HF, patients with a QRS interval of 120–149 ms were required to meet two of three imaging criteria for ventricular dyssynchrony: an aortic pre‐ejection delay of >140 ms, an interventricular mechanical delay of >40 ms, or delayed activation of the posterolateral LV wall. In order to avoid any confounding effect of concomitant defibrillator therapy, patients in COMPANION assigned to CRT‐D were excluded from this meta‐analysis; data comparing CRT‐D to control have already been published.^1^

The following variables, measured at baseline, were merged into a single database: age, sex, New York Heart Association (NYHA) class, ischaemic heart disease (IHD), height, weight, body surface area (BSA), body mass index (BMI), heart rate, systolic blood pressure, QRS intervals, QRS morphology, LVEF, and treatments for heart failure. QRS morphology and duration were recorded by investigators in COMPANION and by a core laboratory in CARE‐HF. For this analysis, QRS duration was classified as <130 ms, 130–139 ms, 140–149 ms, 150–179 ms, 180–199 ms, and ≥200 ms based on the inclusion criteria of landmark trials and guidelines.

### Conduct of the trials

For COMPANION, after 1520 patients had been enrolled, the target number of 1000 primary events was reached, meeting the criteria for trial termination^1^; 1020 patients had a primary event (composite of hospitalization for any reason [other than the one required for device implantation] or all‐cause mortality), 313 died and 594 had a hospitalization for heart failure or died.^1^ For mortality, the median duration of follow‐up was 14.8 months in the control group and 16.0 months in those assigned to CRT‐P; 4% of those assigned to the control group and 1% of those assigned to CRT‐P were lost to follow‐up. A device could not be implanted in 78 of 617 patients assigned to CRT‐P and five deaths were considered procedure related. At least 18 patients assigned to control received a CRT device during follow‐up. Compared to the control group, patients assigned to CRT‐P or CRT‐D had similar and significant reductions in the primary composite endpoint and in the composite of hospitalization for heart failure or all‐cause mortality. Patients assigned to CRT‐P and CRT‐D had similar mortality rates although, compared to the control group, the reduction achieved statistical significance only for CRT‐D.

CARE‐HF was completed according to plan, enrolling 813 patients of whom 383 reached the primary endpoint (composite of unplanned hospitalization for a major cardiovascular event or all‐cause mortality); 202 patients died and 309 had a HF hospitalization or died. The median duration of follow‐up was 30 months (range 18–45) for the main trial and no patient was lost to follow‐up. A device could not be implanted in 19 (4.6%) patients assigned to CRT after one or more attempts. In the control group, 50 patients had either an ICD or CRT device implanted but this occurred in only 19 patients before they had reached the primary endpoint. A further 53 deaths occurred during a 6‐month post‐trial extension phase during which most patients remained on assigned treatment.^2^, ^16^, ^17^ The trial demonstrated lower rates of all‐cause mortality and the composite of hospitalization for heart failure or all‐cause mortality for patients assigned to CRT‐P.

### Statistics

Values given are median and interquartile range for continuous variables, or proportions (percentages) for categorical variables. Baseline characteristics are shown classified by QRS duration and by QRS morphology. The pre‐specified outcomes for this analysis were all‐cause mortality and the composite of hospitalization for heart failure or all‐cause mortality.

The principal analyses were by Cox constant proportional hazards models, including main effects for randomized therapy (CRT‐P or pharmacological treatment) and trial. The influence of a baseline characteristic and outcome was examined by including the item as a main effect and an interaction with randomized therapy. Pre‐specified candidate effect modifiers were examined individually. Multivariable models were developed including those candidate effect modifiers which achieved a significant effect in a univariate model. Items were retained in the multivariable model if they achieved a significant improvement in overall model fit according to the Akaike Information Criterion (AIC). Statistical analyses for events were done using the intention‐to‐treat principle and included patients who failed to receive their assigned treatment, those whose device failed to deliver effective resynchronization and patients in the control group who had a device implanted (‘drop‐ins’).

## Results

Overall, 712 patients were assigned to control and 1026 to CRT‐P. Information on QRS duration was available on 1720 patients and QRS morphology on 1700. Most patients were men (70%), 68% had a QRS duration between 150 ms and 199 ms and 80% had LBBB. LVEF was lower and more patients had IHD in COMPANION. These differences and the requirement for a recent hospitalization might explain the higher annualized rates for hospitalization for heart failure and mortality in COMPANION compared to CARE‐HF.

Stratification by QRS duration (*Table* *1*) found that the proportion with non‐specific interventricular conduction delay (NIVCD) fell from 41% of those with QRS duration <130 ms to 5% for those with QRS duration >150 ms. About 10% of patients had right bundle branch block (RBBB). Patients with shorter QRS durations were more likely to be women and less likely to have IHD. LVEF declined as QRS duration increased. Pharmacological management was broadly similar for different QRS durations. Stratification by QRS morphology (*Table* *2*) showed that patients with LBBB were more likely to be women and less likely to have IHD.

**Table 1:** Patient characteristics according to QRS duration

	Overall	QRS duration					
		<130 ms	130–139 ms	140–149 ms	150–179 ms	180–199 ms	>200 ms
Patients, *n*	1720	165	92	166	890	271	136
Age, years	67 (59–73)	66 (55–73)	65 (56–72)	67 (59–74)	67 (59–73)	67 (60–74)	67 (61–72)
Women	509 (30)	37 (22)	27 (29)	44 (27)	302 (34)	63 (23)	36 (26)
IHD	948 (55)	51 (31)	37 (40)	62 (37)	414 (47)	146 (54)	62 (46)
NYHA class IV	182 (11)	23 (14)	8 (9)	19(11)	89 (10)	23 (8)	20 (15)
Height, cm	170 (163–178)	173 (165–180)	172 (164–180)	172 (165–178)	170 (162–176)	173 (166–178)	170 (163–177)
Weight, kg	82 (70–97)	82 (70–97)	80 (70–95)	83 (69–96)	77 (67–90)	80 (70–89)	78 (65–91)
BMI, kg/m^2^	26.8 (23.8–30.7)	27.9 (23.7–32.6)	27.6 (24.5–31.8)	27.2 (23.8–31.9)	26.8 (23.9–30.4)	26.3 (23.2–29.8)	26.5 (23.1–30.1)
BSA, m^2^	1.94 (1.77–2.10)	1.99 (1.83–2.14)	1.95 (1.79–2.18)	1.99 (1.79–2.14)	1.91 (1.74–2.07)	1.96 (1.79–2.09)	1.92 (1.76–2.10)
Heart rate, bpm	70 (62–80)	72 (63–80)	75 (63–80)	75 (63–82)	71 (62–80)	68 (60–77)	66 (60–76)
Systolic BP, mmHg	114 (102–128)	112 (100–128)	110 (100–123)	112 (102–130)	115 (104–130)	112 (104–125)	110 (100–120)
PR interval, ms	200 (178–216)	190 (172–212)	200 (180–220)	188 (164–212)	196 (175–210)	200 (184–220)	200 (190–230)
LBBB	1369 (80)	90 (54)	54 (59)	117 (70)	756 (86)	235 (88)	117 (88)
RBBB	139 (8)	8 (5)	14 (15)	16 (10)	73 (8)	17 (6)	11 (8)
NIVCD	190 (11)	67 (41)	24 (26)	33 (20)	47 (5)	14 (5)	5 (4)
LVEF	24 (20–29)	25 (20–30)	25 (20–30)	24 (20–30)	24 (20–28)	22 (18–26)	20 (17–25)
ACEi/ARB	1574 (92)	137 (83)	86 (93)	155 (93)	821 (92)	255 (94)	120 (88)
Beta‐blocker	1199 (70)	123 (75)	63 (68)	115 (69)	617 (69)	187 (69)	94 (69)
MRA	949 (55)	92 (56)	50 (54)	89 (54)	498 (56)	147 (54)	73 (54)

Values are *n* (%) or median (first–third quartile).

ACEi, angiotensin‐converting enzyme inhibitor; ARB, angiotensin receptor blocker; BMI, body mass index; BP, blood pressure; BSA, body surface area; IHD, ischaemic heart disease; LBBB, left bundle branch block; LVEF, left ventricular ejection fraction; MRA, mineralocorticoid receptor antagonist; NIVCD, non‐specific inter‐ventricular conduction delay; NYHA, New York Heart Association; RBBB, right bundle branch block.

**Table 2:** Patient characteristics according to QRS morphology

	QRS morphology		
	LBBB	RBBB	NIVCD
Patients, *n*	1371*	139	190
Age, years	67 (59–73)	69 (61–75)	64 56 72
Women	449 (33)	18 (13)	38 (20)
IHD	693 (51)	115 (83)	125 (66)
NYHA class IV	131 (10)	19 (14)	28 (15)
Height, cm	170 (163–178)	174 (168–178)	173 (168–180)
Weight, kg	78 (67–90)	80 (72–92)	83 (73–96)
BMI, kg/m^2^	26.7 (23.7–30.6)	26.8 (24.0–29.9)	28.1 (23.9–32.6)
BSA, m^2^	1.92 (1.75–2.09)	1.95 (1.83–2.13)	2.01 (1.88–2.14)
Heart rate, bpm	70 (62–80)	74 (62–80)	73 (64–80)
Systolic BP, mmHg	115 (104–130)	110 (100–122)	110 (100–122)
PR interval, ms	198 (176–212)	201 (184–236)	200 (178–220)
QRS duration, ms	160 (150–180)	160 (148–175)	140 (124–156)
LVEF (%)	23.6 (20.0–28.1)	25.0 (20.0–30.0)	25.0 (20.0–30.0)
ACEi/ARB	1271 (93)	116 (83)	170 (89)
Beta‐blocker	984 (72)	75 (54)	127 (67)
MRA	756 (55)	73 (53)	107 (56)

Values are *n* (%) or median (first–third quartile).

ACEi, angiotensin‐converting enzyme inhibitor; ARB, angiotensin receptor blocker; BMI, body mass index; BP, blood pressure; BSA, body surface area; IHD, ischaemic heart disease; LBBB, left bundle branch block; LVEF, left ventricular ejection fraction; MRA, mineralocorticoid receptor antagonist; NIVCD, non‐specific inter‐ventricular conduction delay; NYHA, New York Heart Association; RBBB, right bundle branch block.

Note: Two patients were reported to have LBBB but QRS duration was missing.

Patients assigned to CRT‐P had a lower risk of all‐cause mortality (hazard ratio [HR] 0.68, 95% confidence interval [CI] 0.56–0.81; *p* < 0.0001) and the composite outcome (HR 0.67, 95% CI 0.58–0.78; *p* < 0.0001) (*Graphical Abstract*).

**Figure 1:**
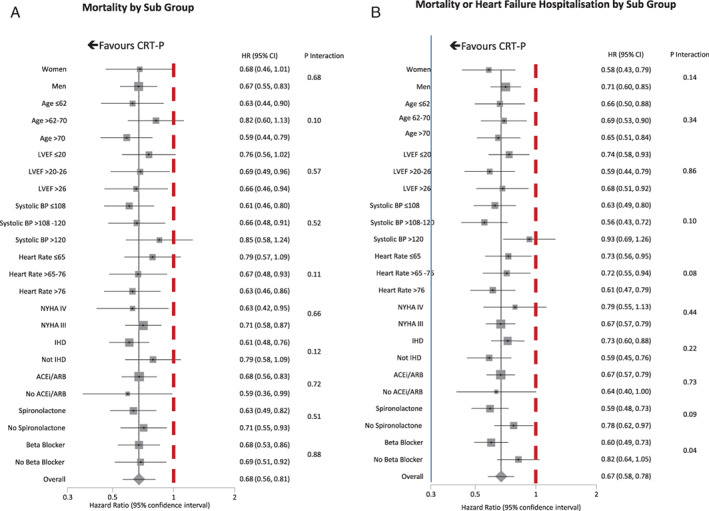
(*A*) Effect of cardiac resynchronization therapy pacemaker (CRT‐P) on all‐cause mortality in clinical subgroups. (*B*) Effect of CRT‐P on heart failure hospitalization or death in clinical subgroups. ACEi, angiotensin‐converting enzyme inhibitor; ARB, angiotensin receptor blocker; BP, blood pressure; CI, confidence interval; HR, hazard ratio; IHD, ischaemic heart disease; LVEF, left ventricular ejection fraction; NYHA, New York Heart Association.

**Figure 2:**
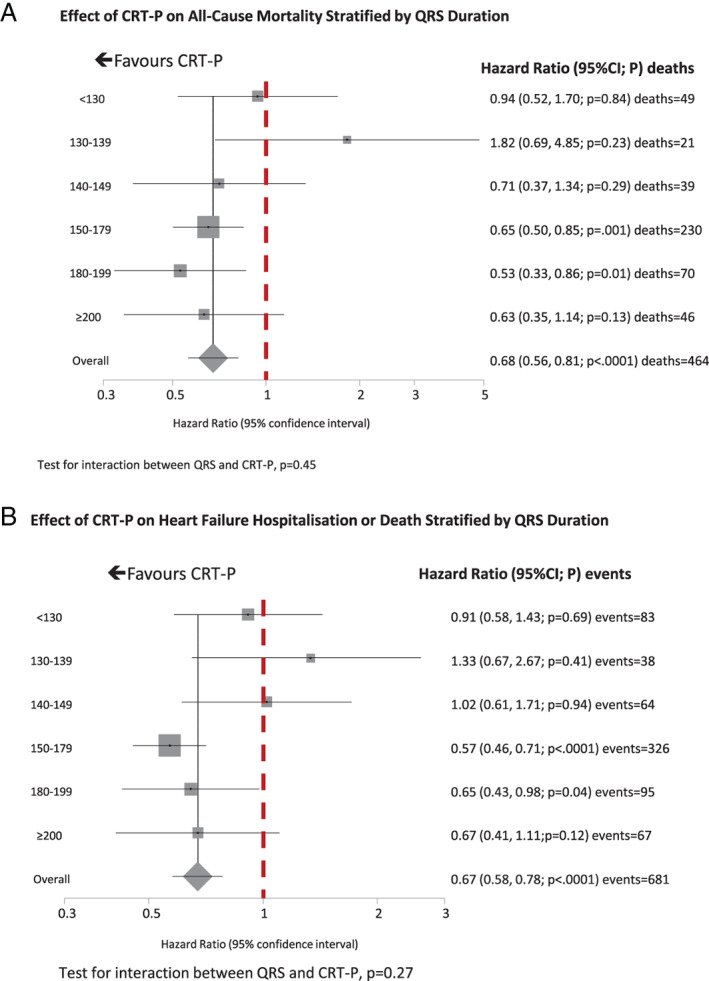
(*A*) Effect of cardiac resynchronization therapy pacemaker (CRT‐P) on all‐cause mortality stratified by QRS duration. Test for heterogeneity *p* = 0.104. (*B*) Effect of CRT‐P on heart failure hospitalization or death stratified by QRS duration. Test for heterogeneity *p* = 0.269. CI, confidence interval.

**Figure 3:**
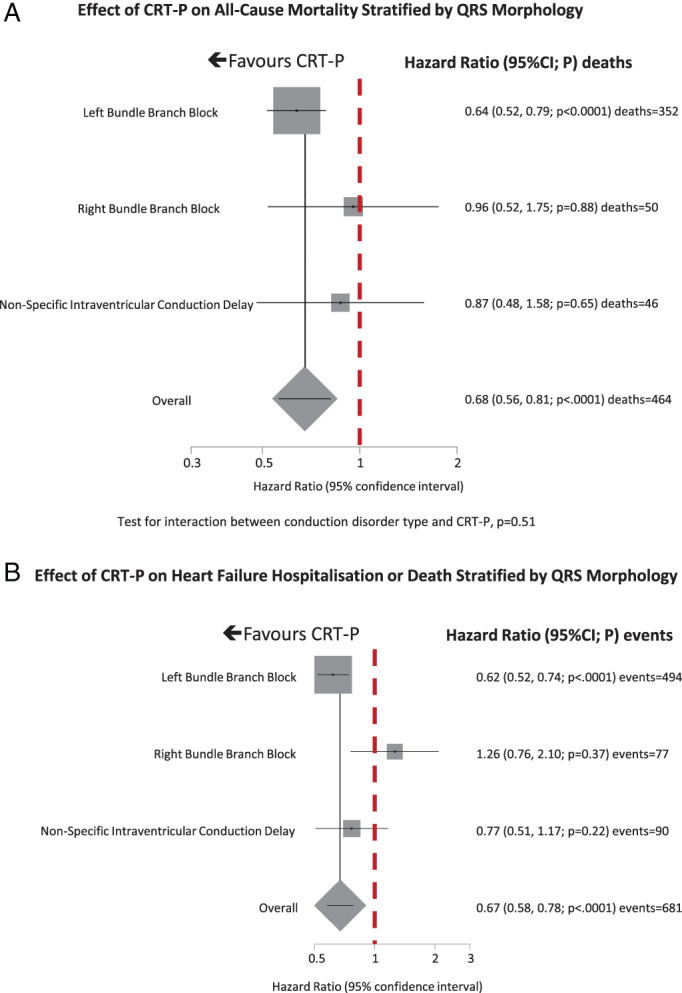
(*A*) Effect of cardiac resynchronization therapy pacemaker (CRT‐P) on all‐cause mortality stratified by QRS morphology. Test for heterogeneity *p* = 0.506. (*B*) Effect of CRT‐P on heart failure hospitalization or death stratified by QRS morphology. Test for heterogeneity *p* = 0.089. CI, confidence interval.

**Figure 4:**
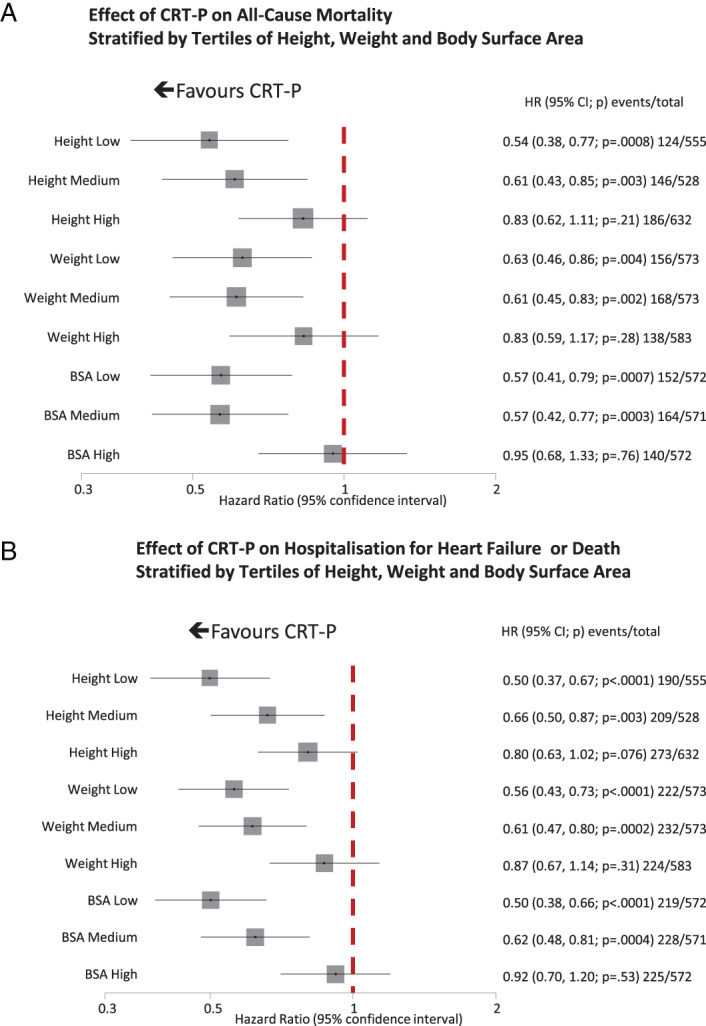
(*A*) Effect of cardiac resynchronization therapy pacemaker (CRT‐P) on all‐cause mortality stratified by height, weight and body surface area (BSA) tertiles. Tests for heterogeneity *p* = 0.128, 0.148 and 0.139, respectively. (*B*) Effect of CRT‐P on heart failure hospitalization or death stratified by height, weight and BSA tertiles. Tests for heterogeneity *p* = 0.028, 0.039 and 0.027, respectively. CI, confidence interval; HR, hazard ratio.

Subgroup analysis failed to identify heterogeneity in the effect of CRT‐P on all‐cause mortality (*Figures* *1*, *2*, *3*, *4*). On univariate analysis, point estimates suggested that CRT‐P might be less likely to reduce mortality in patients with QRS duration <140 ms but the CIs were wide (*Figure* *2*). Univariate point estimates also suggested that CRT‐P might be less likely to reduce mortality in patients with RBBB or NIVCD, but the CIs entirely overlapped those for LBBB (*Figure* *3*). CRT‐P also tended to have a greater effect on mortality in patients of smaller stature (*Figure* *4*).

For the composite outcome, the subgroup analysis produced broadly similar results (*Figures* *2*, *3*, *4*). However, for QRS morphology, the HR for RBBB exceeded 1.0 and the 95% CI did not overlap that for the HR for LBBB (*Figure* *3*). CRT‐P had a greater effect in patients prescribed beta‐blockers, with a similar trend for those prescribed spironolactone but an interaction with angiotensin‐converting enzyme (ACE) inhibitors or angiotensin receptor blockers was not observed. CRT‐P also had a greater effect in patients of smaller stature, whether classified by tercile of height, weight or BSA (*Figure* *4*), but not by BMI, a measure of adiposity (*p* for interaction = 0.33). Neither was the effect of CRT‐P on mortality influenced by BMI (*p* for interaction = 0.55). In order to exclude the possibility that BSA was acting as a surrogate for sex, the analysis was repeated solely for men (*Table* *3*), which showed broadly similar results, although no longer statistically significant.

**Table 3:** Multivariable model developed from *Table* *4* showing independent predictors for the composite endpoint of hospitalization for heart failure or death for patients of both sexes and for men alone

	Women and men			Only men		
	HR	95% CI	*p*‐value	HR	95% CI	*p*‐value
Enrolled in CARE‐HF	0.599	0.510–0.704	<0.0001	0.527	0.436–0.637	<0.0001
Being assigned to CRT‐P	0.188	0.058–0.608	0.0053	0.217	0.045–1.047	0.057
BSA	0.631	0.405–0.983	0.0417	0.563	0.311–1.021	0.0588
CRT‐P*BSA	2.169^a^	1.187–3.967	0.0119	1.954^a^	0.888–4.300	0.0961
Beta‐blocker	0.791	0.630–0.994	0.0439	0.802	0.615–1.046	0.1038

BSA, body surface area; CI, confidence interval; CRT‐P, cardiac resynchronization therapy pacemaker; HR, hazard ratio.

Patients enrolled in the CARE‐HF trial had a lower mortality (regardless of assigned group) than patients enrolled in COMPANION. Patients with lower BSA and/or receiving beta‐blockers had a significantly greater benefit from CRT. Although *p*‐values do not quite achieve conventional levels of significance when the analysis is confined to men, the results are similar to those of the overall population.

^a^ Less benefit from CRT‐P with higher BSA.

No statistically significant variation in the reduction in mortality with CRT‐P was observed for any pre‐specified variable (*Table* *4*); accordingly, a multivariable model was not attempted. However, for the composite outcome, CRT‐P had a greater effect on patients with a lower BSA and those prescribed a beta‐blocker (*Table* *4*), effects that were also observed in a multivariable model (*Table* *3*). Patients prescribed beta‐blockers were younger, more likely to have LBBB and were less likely to be in NYHA class IV or have IHD.

**Table 4:** Univariate analysis of the association between patient characteristics and the effects of CRT‐P on mortality and on the composite endpoint of hospitalization for heart failure or death

Characteristic	Mortality			Composite endpoint		
	HR	95% CI	*p*‐value	HR	95% CI	*p*‐value
Age	0.984	0.966–1.003	0.102	0.993	0.978–1.008	0.340
Female sex	0.913	0.591–1.411	0.682	0.769	0.544–1.089	0.140
IHD	0.739	0.504–1.083	0.121	1.217	0.890–1.665	0.219
NYHA class III	1.109	0.703–1.748	0.657	0.856	0.577–1.270	0.439
Height	1.015	0.996–1.035	0.128	**1.018**	**1.002–1.034**	**0.028**
Weight	1.008	0.997–1.018	0.148	**1.009**	**1.000–1.017**	**0.039**
BSA	1.011	0.976–1.047	0.553	**1.968**	**1.081–3.584**	**0.027**
BMI	1.733	0.836–3.589	0.139	1.014	0.986–1.044	0.326
Heart rate	0.989	0.975–1.003	0.114	0.990	0.978–1.001	0.081
Systolic BP	1.004	0.992–1.015	0.523	1.008	0.999–1.017	0.097
PR interval	1.001	0.997–1.006	0.585	1.000	0.996–1.004	0.978
QRS duration	0.993	0.985–1.001	0.104	0.996	0.989–1.003	0.269
QRS morphology			0.506			0.089
LVEF (%)	0.992	0.963–1.021	0.574	1.002	0.979–1.026	0.858
ACEi/ARB	0.907	0.531–1.547	0.719	0.920	0.571–1.483	0.733
Beta‐blockers	0.973	0.671–1.410	0.884	**0.717**	**0.526–0.977**	**0.035**
MRA	0.884	0.613–1.273	0.507	0.770	0.569–1.043	0.092

ACEi, angiotensin‐converting enzyme inhibitor; ARB, angiotensin receptor blocker; BMI, body mass index; BP, blood pressure; BSA, body surface area; CI, confidence interval; HR, hazard ratio; IHD, ischaemic heart disease; LVEF, left ventricular ejection fraction; MRA, mineralocorticoid receptor antagonist; NYHA, New York Heart Association.

## Discussion

This IPD analysis confirms the substantial reduction in mortality exerted by CRT‐P, in addition to pharmacological therapy, for appropriately selected patients with heart failure. Most patients had a QRS duration exceeding 140 ms and LBBB and therefore it is for such patients that the benefits of CRT are most certain. In common with two previous IPD meta‐analyses, we failed to show that the effects of CRT differ according to QRS morphology.^3^, ^4^ However, in contrast to previous analyses, an independent relationship between QRS duration and the benefits of CRT was not observed.^3^, ^4^ Failure to demonstrate an interaction with either QRS morphology or duration may reflect the small number of patients in some subgroups. Although point estimates of the effects of CRT in patients with QRS duration <140 ms or without LBBB appeared less favourable, the wide CIs around these observations do not exclude similar benefits regardless of QRS morphology or duration, provided it is at least 120 ms. This is particularly the case for mortality, arguably the more robust measure of treatment effect.

The mechanisms by which CRT exerts its benefits remain uncertain (*Graphical Abstract*). Some suggest that the severity of LV dyssynchrony and of the delay in LV free wall activation should predict the benefits of CRT.^18^ However, clinical trials have failed to show a convincing relationship between such abnormalities of ventricular function and outcomes.^5^, ^18^ Improvement in inter‐ventricular or atrio‐ventricular dyssynchrony, reductions in systolic and diastolic mitral regurgitation, beneficial LV remodelling, reductions in tachy‐arrhythmias and prevention of pauses provide alternative mechanisms for the benefits of CRT.^19^ All of these mechanisms may be important for the actions of CRT but might vary amongst individuals and over time or with physical activity. Although LV function often improves immediately with CRT^20^ and with longer‐term LV remodelling,^21^ this may be a poor surrogate for the clinical benefits of CRT because, despite smaller improvements in LV function for patients with IHD,^21^, ^22^ the effect of CRT on morbidity and mortality is remarkably similar regardless of the aetiology of left ventricular dysfunction.^3^, ^4^

None of the landmark trials used QRS morphology as an inclusion criterion, but guidelines both on the prevention of sudden death and on heart failure give stronger recommendations for patients who have LBBB, despite the lack of evidence from pre‐specified subgroup analyses.^6^, ^7^, ^8^, ^9^ Observational studies suggest that patients with RBBB have a worse outcome, but this may reflect their higher prevalence of IHD rather than less response to CRT.^23^ Moreover, many patients with RBBB have concealed LBBB, blurring the distinction between groups.^5^, ^24^, ^25^ However, the paucity of data for patients with a QRS morphology other than LBBB creates uncertainty and provides some justification for a lower strength of guideline recommendations for patients who do not have LBBB.

Although point estimates for the HRs for mortality or the composite endpoint suggested less benefit when QRS duration was <140 ms, we did not observe a statistically significant relationship between QRS duration and the effects of CRT in this analysis. This may reflect the small number of patients with shorter QRS durations. However, a previous IPD meta‐analysis^3^ and the Echo‐CRT trial suggest that CRT might be harmful in patients with a QRS duration <130 ms.^26^ Thus, in contrast to QRS morphology, data from several sources support QRS duration as an important independent predictor of the benefits of CRT.

Patients treated with beta‐blockers appeared to benefit more from CRT, at least for the composite outcome, as previously shown.^27^ This could reflect synergy between two interventions that exert powerful effects on ventricular remodelling. CRT may also facilitate titration to higher doses of beta‐blockers by increasing systolic blood pressure and by allaying fear of bradycardia. However, those not prescribed beta‐blockers had characteristics indicating an intrinsically worse prognosis. Patients treated with spironolactone also tended to benefit more from CRT but similar trends were not apparent for ACE inhibitors or angiotensin receptor blockers. Neither sacubitril/valsartan nor sodium–glucose cotransporter inhibitors were available when these trials of CRT‐P were being conducted. However, it is likely that the benefits of CRT‐P and these newer interventions are complementary.^28^

Some analyses suggest that women obtain more benefit from CRT than men.^29^ We did not find this. However, sex is strongly associated with height, weight and, therefore, BSA. We observed an interaction between the effects of CRT and BSA for the composite outcome with smaller patients obtaining greater benefit from CRT, as observed in a previous IPD meta‐analysis for both morbidity and mortality.^30^ No interaction was observed with BMI, an index of adiposity rather than body size.^31^ It is uncertain why patients with higher BSA should obtain less benefit. It does not appear to be a surrogate for sex because the observed benefit was similar in men and women of similar size but this possibility cannot be entirely discounted. People with a smaller BSA might have an inherently shorter QRS^32^ and smaller ventricular dimensions. A QRS duration of 140 ms may be markedly prolonged for a short person but not for a tall one. It is also possible that having LV dilatation as an inclusion criterion for these trials meant that smaller patients had to have more severe LV dysfunction to be included. The incidence of atrial fibrillation, which may reduce the effectiveness of CRT,^33^, ^34^ also increases with height^35^ and its genetic determinants.^36^ Perhaps body size should be considered when applying QRS criteria for CRT for populations of smaller stature than those in Europe and North America, where these trials were done.^37^

Most trials compared CRT to another device. This was done either to permit blinding and minimize bias when assessing subjective measures of response to CRT^10^, ^38^ or to investigate the incremental benefit of CRT over ICD alone.^3^, ^4^ This strategy probably improved recruitment, since patients are more likely to agree to participate if they are going to receive a device anyway and, as health services pay for clinically‐indicated ICDs, this helped defray costs. Trials comparing devices provide evidence for which device to select but not whether the patient should have a device in the first place. The effects of ICD and CRT may not be additive.^4^ Implanting an ICD in the control group could deliver some of the benefits of CRT, such as prevention of pauses, or could be harmful, by inducing dyssynchrony through right ventricular pacing. Proof that CRT‐D is superior to CRT‐P is lacking but the acquisition cost and complications certainly differ.^39^ A trial comparing CRT‐P and CRT‐D is ongoing (RESET‐CRT, NCT03494933) and others are planned.

In conclusion, this IPD analysis confirms the benefits of CRT‐P on morbidity and mortality. Patients with a smaller body size and those receiving beta‐blockers might gain greater benefit. However, robust evidence is lacking that either QRS duration within the studied range or QRS morphology are independent predictors of the benefits of CRT‐P.

### Funding

John G.F. Cleland is supported by a British Heart Foundation Centre of Research Excellence award RE/18/6/34217.

This analysis was done without financial support from industry. Representatives from Medtronic and Boston Scientific, who funded the original trials, facilitated data sharing, were shown the results and were allowed to comment.

**Conflict of interest:** J.G.F.C.: grants and speaker honoraria from Amgen, Medtronic and Novartis. N.F.: research and consulting for Sanofi Aventis, Novo Nordisk, Takeda, Allergan, Ipsen, AstraZeneca, Accelovance PCT. B.O.: Amarin (chair DSMB), Lundbeck (speaker and consultant), Boehringer Ingelheim (honoraria). D.G.: consulting for Medtronic, Boston Scientific, Abbott (St Jude), Biotronik. L.S.: consulting for Boston Scientific. J.B.: consulting for Boston Scientific, Medtronic and St. Jude Medical/Abbott. A.M.F.: equity in Renovacor. All other authors have nothing to disclose.
